# Relationship between Serum Carotenoids and Hyperglycemia: a Population-based Cross-sectional Study

**DOI:** 10.2188/jea.12.357

**Published:** 2007-11-30

**Authors:** Koji Suzuki, Yoshinori Ito, Sayuri Nakamura, Junichi Ochiai, Kunio Aoki

**Affiliations:** 1 Department of Public Health, Fujita Health University School of Health Sciences.; 2 Department of Adult Nursing, Fujita Health University School of Health Sciences.; 3 Department of Medical Electronics, Fujita Health University School of Health Sciences.; 4 Aichi Cancer Center.

**Keywords:** intake of fruit and vegetables, carotenoids, beta-carotene, hyperglycemia, diabetes mellitus

## Abstract

The present study investigated the relationship between hyperglycemia and both serum carotenoids and intake of vegetables and fruits. Subjects with a history of diabetes mellitus (DM group, n=133) or with hyperglycemia diagnosed using a 5.6% cutoff value for hemoglobin A_1c_ (High HbA_1C_ group, n=151) were recruited from among inhabitants of a rural area in Hokkaido, Japan. Intake frequencies of vegetables and fruits were assessed using a questionnaire administered by public health nurses. Serum levels of carotenoids and retinol were measured using high-performance liquid chromatography. The relationships between high HbA_1C_ or DM and both serum carotenoids and intake frequencies of vegetables and fruits were analyzed using logistic regression modeling for a case-control study; each case (High HbA_1C_ or DM) was matched to two controls (healthy subjects without any history of disease) matched for sex and age (within 3 years). The odds ratio (OR) for high HbA_1C_ was 0.49 (95% confidence interval: 0.29-0.85) on high intake frequency of carrot and pumpkin and the OR for DM was 1.21 (95% CI: 0.79-1.84). No significant relationships were observed between high HbA_1c_ and intake frequencies of other vegetables and fruits. The ORs on high serum levels of *α*- and *β*-carotenes, lycopene, *β*-cryptoxanthin and zeaxanthin and lutein were 0.38 (0.22-0.65), 0.35 (0.21-0.59), 0.57 (0.35-0.93), 0.35 (0.20-0.59), and 0.88 (0.54-1.46) for high HbA_1c_, respectively. In conclusion, intake of vegetables and fruits rich in carotenoids might be a protective factor against hyperglycemia.

Diabetes mellitus (DM) is characterized by chronic hyperglycemia due to insufficiency of insulin. Chronic hyperglycemia leads to complications such as retinopathy, nephropathy, and neuropathy.^1^ In addition, DM is a risk factor for coronary heart disease and cerebrovascular disease.^2^ In Japan, the prevalence of diabetes among those aged 40 years and older is estimated to be 6-7%^3^ and 6.3 million DM patients were identified in 1995.^3^ The number of DM patients in Japan is estimated to reach 8.5 million in 2025.^4^

Hyperglycemia increases the generation of free radicals by glucose autoxidation and increases levels of the glycoprotein Hemoglobin A_1c_ (HbA_1c_).^1^^,^^5^^-^^7^ Hyperglycemia enhances oxidation stress such as increasing serum lipid peroxidation.^1^^,^^7^^,^^8^ The relationship between pathogenesis of DM and oxidation stress is unclear, but a close relation exists between the pathogenesis of DM complications and tissue injury from free radicals.^9^

Carotenoids such as *β*-carotene, contained in fruit and vegetables, protect cells from oxidative stress by quenching free radicals.^9^^,^^10^ Numerous epidemiologic studies have demonstrated that subjects with higher dietary consumption of fruit and vegetables rich in carotenoids, or with high serum level of *β*-carotene have lower risks of certain cancers and cardiovascular disease.^9^^,^^11^^,^^12^ A recent study^13^ found that serum levels of *β*-carotene and lycopene were lower among Americans with DM than those without. However, few reports have investigated serum carotenoids levels of subjects with chronic hyperglycemia, a pre-DM condition. HbA_1c_ is a biomarker of long-term glucose homeostasis that reflects blood glucose concentrations over the previous 1 or 2 months.^14^ High HbA_1c_ concentrations indicate a condition of chronic hyperglycemia.^14^

In the present study, we investigated the relationship between high HbA_1c_ or DM and intake frequencies of fruit and vegetables and serum carotenoids levels in rural Japanese subjects.

## SUBJECTS AND METHODS

Health examinations of inhabitants aged 40 years or older (range: 40 to 86 years) have been performed in an area of Hokkaido, Japan, every August since 1982.^12^ The population for this study comprised 1,691 subjects (656 males and 1,035 females) who attended the health examinations from 1998 through 2000. We used the first data as to repeaters. Of these, 151 subjects (81 males and 70 females) displayed HbA_1c_ values of 5.6% or more and no history of DM (High HbA_1c_ group). Other 133 subjects (73 males and 60 females) displayed a history of DM (DM group).

We randomly selected two controls for each subject in the High HbA_1c_ group (Healthy 1 group) and DM group (Healthy 2 group) from health examination participants with no history of illness such as apoplexy, myocardial infarction, angina pectoris, hypertension, liver disease, renal disease, gout, DM, or cancer. Control subjects were pair-matched by sex and age (within 3 years).

Trained public health nurses administered a questionnaire regarding health and daily lifestyle habits, assessing smoking (current smoker, ex-smoker, or non-smoker), alcohol consumption (regular drinker, ex-drinker, or non-drinker), dietary intake of major foods (rarely, 1-2 times/month, 1-2 times/week, 3-4 times/week, or almost daily), physical exercise (rarely, 1-2 hours/week, 3-4 hours/week, or 5+ hours/week), and histories of major illnesses at the time of the health examination.

Fasting serum samples were taken during the health examinations and sera were separated from blood cells by centrifugation within 1 hour. Biochemical analysis of the sampled sera was performed using an auto-analyzer (JCA-RX20, Nihon Denshi Co. Ltd.). Whole blood HbA_1c_ was measured by high-performance liquid chromatography.^15^

Serum levels of *α*- and *β*-carotene, lycopene, *β*-cryptoxanthin, zeaxanthin and lutein (zeaxanthin & lutein), canthaxanthin, retinol, and *α*-tocopherol were measured separately by high-performance liquid chromatography.^16^

Serum level of thiobarbituric acid-reactive substance (TBARS) was determined using the thiobarbituric acid reaction method.^17^

Body height, weight and blood pressure were measured during the health examinations. Body mass index (BMI) was calculated as body weight (kg) divided by height (m) squared.

We obtained informed consent with the participants’ signatures for providing information and serum to our epidemiologic study.

All statistical analyses, such as t tests, chi-square tests, and logistic regression analyses, were conducted using the statistical package (Stat View ver.5.0). To assess the relationship between intake frequencies of fruit and vegetables and the risks of high HbA1c and DM, intake frequency of fruit and vegetables were divided into two groups (low: 1-2 times/week or less, high: 3-4 times/week or more), and logistic regression analyses were performed after adjusting for smoking and alcohol consumption. To assess the relationship between serum levels of carotenoids and the risks of high HbA_1c_ and DM, serum levels of carotenoids were divided into three groups (low, middle, and high) according to the distribution of serum carotenoids levels in Healthy 1 or 2 groups, and logistic regression analyses were performed after controlling for smoking habit and alcohol consumption, systolic blood pressure, BMI, and serum levels of triglyceride, total cholesterol, and *γ*-GTP activity. These potential confounding factors were strongly associated with serum carotenoids levels. Serum levels of carotenoids such as *β*-carotene are lower in smoker or alcohol drinker.^18^ Serum *β*-carotene levels were negatively associated with systolic blood pressure and BMI,^18^^,^^19^ and positively associated with serum levels of total cholesterol and triglyceride.^19^^,^^20^ Serum carotenoids levels were also affected by liver function, because absorbed carotenoids are stored in several organs, especially in the liver and adipose tissue.^21^ Serum level of *γ*-GTP activity, a biomarker of liver function, was added in adjusting factors.

## RESULTS

Table 1 shows the characteristics of subjects in this study. The percentage of current alcohol drinkers was significantly higher in the High HbA_1c_ group than in the Healthy 1 group. No significant differences in smoking, alcohol consumption, or exercise were observed between the DM and Healthy 2 groups.

**Table 1.** Characteristics of the subjects

Items	High HbA_1c_		Healthy 1			p	DM				Healthy 2					p
No .^a^	151	(100.0)	302		(100.0)		133			(100.0)	266			(100.0)		
male	81	(53.6)	162		(53.6)	n.s.^$^	73			(54.9)	146			(54.9)		n.s.^$^
female	70	(46.4)	140		(46.4)		60			(45.1)	120			(45.1)		

Age (year)^b^	63.6 ± 9.9		63.1 ± 9.4			n.s.^#^	63.8 ± 9.7				63.7 ± 9.61					n.s.^#^

Smoking habit ^a^																
never smoker	74	(49.0)	170	(56.3)		n.s.^$^	63	(47.4)			142		(53.4)			n.s.^$^
ex-smoker	35	(23.2)	51	(16.9)			34	(25.6)			54		(20.3)			
current smoker	42	(27.8)	81	(26.8)			36	(27.1)			70		(26.3)			

Alcohol consumption ^a^																
non-drinker	79	(52.3)	206	(68.2)		***^$^	77	(57.9)			183		(68.8)			n.s.^$^
ex-drinker	7	(4.6)	7	(2.3)			17	(12.8)			8		(3.0)			
regular drinker	64	(42.4)	87	(28.8)			39	(29.3)			74		(27.8)			

Physical exercise ^a^																
none	100	(66.2)	207	(68.5)		n.s.^$^	80		(60.2)		179		(67.3)			n.s.^$^
1-2 hours/week	27	(17.9)	43	(14.2)			20		(15.0)		35		(13.2)			
3-4 hours/week	9	(6.0)	25	(8.2)			14		(10.5)		25		(9.4)			
5+ hours/week	13	(8.6)	27	(8.9)			16		(12.0)		25		(9.4)			

High HbA_1c_: the subjects with no history of diabetes mellitus with HbA_1c_ values of 5.6% or more.

DM: the subjects with history of diabetes mellitus

Healthy1: the healthy control group for High HbA_1c_ group, Healthy2: the healthy control group for DM group.

^a^ : The number and percentage in parenthesis.

^b^ : mean ± standard deviation.

^#^: t test,^$^ : chi-square test, ***: p<0.001

Table 2 shows the levels of HbA_1c_, serum components, blood pressure, and BMI among the study groups. The levels of HbA_1c_, serum fasting glucose, triglyceride, TBARS, *γ*-GTP, systolic blood pressure, diastolic blood pressure and BMI were significantly higher in the High HbA_1c_ group than in the Healthy 1 group. HDL-cholesterol was significantly lower in the High HbA1c group than in the Healthy 1 group. HbA_1c_, serum fasting glucose, triglyceride, *γ*-GTP, systolic blood pressure and BMI were significantly higher in the DM group than in the Healthy 2 group. HDL-cholesterol was significantly lower in the DM group than in the Healthy 2 group. HbA_1c_ was significantly higher and serum total cholesterol, systolic blood pressure and diastolic blood pressure were significantly lower in the DM group than in the High HbA_1c_ group.

**Table 2.** Comparison of serum components, HbA_1c_, blood pressure and BMI among the study groups

Items		High HbA_1c_	Healthy 1	p	DM	Healthy 2	p
Fasting glucose ^a^	(mg/dl)	111.6 ± 34.4	94.2 ± 8.3	***	120.1 ± 38.5	94.3 ± 8.4	***

HbA_1c_^a^	(%)	6.0 ± 0.8	5.0 ± 0.3	***	6.4 ± 1.2	5.0 ± 0.3	***

Total cholesterol ^a^	(mg/dl)	220.2 ± 38.1	215.5 ± 37.1		209.0 ± 31.6	210.4 ± 33.9	

HDL-cholesterol ^a^	(mg/dl)	55.0 ± 15.7	59.0 ± 16.3	*	55.6 ± 15.5	58.9 ± 17.5	*

Triglyceride ^b^	(mg/dl)	123.3	97.5	***	117.9	91.7	***
		(66.6-264.2)	(54.0-186.3)		(64.6-234.4)	(52.0-173.9)	

TBARS ^b^	(nmol/ml)	3.17	2.91	**	3.08	2.90	
		(2.40-3.82)	(2.30-3.70)		(2.45-3.80)	(2.20-3.60)	

*γ*-GTP ^b^	(IU/l)	28.6	18.7	***	26.4	18.2	***
		(12.0-85.4)	( 9.0-51.0)		(11.0-72.2)	(10.0-46.9)	

SBP ^a^	(mmHg)	145.9 ± 20.7	133.8 ± 19.2	***	141.4 ± 18.7	134.9 ± 20.4	**

DBP ^a^	(mmHg)	85.4 ± 10.7	81.4 ± 10.1	***	81.0 ± 11.6	80.8 ± 9.7	

BMI ^a^	(kg/m^2^)	24.8 ± 3.6	23.5 ± 2.9	***	24.5 ± 3.3	23.2 ± 2.8	***

High HbA_1c_: the subjects with no history of diabetes mellitus with HbA_1c_ values of 5.6% or more.

DM: the subjects with history of diabetes mellitus.

Healthy1: the healthy control group for High HbA_1c_ group, Healthy2: the healthy control group for DM group.

TBARS: tiobarbituric acid-reactive substances

SBP: systolic blood pressure, DBP: diastolic blood pressure, BMI: body mass index (Weight / [Hight] ^2^)

^a^ : mean ± standard deviation.

^b^ : geometric mean and range of 10-90% in parenthesis.

p: t test (*:p<0.05, **: p<0.01, ***: p<0.001)

Table 3 shows intake frequency of fruit and vegetables among the study groups. The percentage of subjects with high intake frequency of carrot and pumpkin was significantly lower, and that of tomato tended to be lower in the High HbA_1c_ group than in the Healthy 1 group. The percentage of subjects with intake frequency of oranges over 3-4 times/week tended to be lower in the DM group than in the Healthy 2 group.

**Table 3.** Comparison of intake frequency of vegetables or fruits between the study groups.

Intake frequency of vegetables or fruits		High HbA_1c_		Healthy 1		p	DM		Healthy 2		p
Green leaf vegetables	Low	78	(51.7%)	146	(48.7%)		65	(48.9%)	134	(51.1%)	
	High	73	(48.3%)	154	(51.3%)		68	(51.1%)	128	(48.9%)	

Carrot or Pumpkin	Low	101	(67.8%)	176	(58.3%)	*	76	(57.1%)	164	(61.7%)	
	High	48	(32.2%)	126	(41.7%)		57	(42.9%)	102	(38.3%)	
				
Tomato	Low	82	(55.4%)	141	(46.8%)	#	72	(54.1%)	132	(49.8%)	
	High	66	(44.6%)	160	(53.2%)		61	(45.9%)	133	(50.2%)	

Cabbage or Lettuce	Low	69	(45.7%)	134	(44.5%)		58	(43.6%)	117	(44.0%)	
	High	82	(54.3%)	167	(55.5%)		75	(56.4%)	149	(56.0%)	

Chinese cabbage	Low	116	(77.3%)	232	(77.6%)		105	(80.2%)	197	(74.9%)	
	High	34	(22.7%)	67	(22.4%)		26	(19.8%)	66	(25.1%)	

Seaweeds	Low	67	(45.0%)	155	(51.8%)		68	(52.3%)	131	(49.8%)	
	High	82	(55.0%)	144	(48.2%)		62	(47.7%)	132	(50.2%)	

Oranges	Low	117	(80.1%)	227	(77.2%)		113	(86.9%)	205	(79.2%)	#
	High	29	(20.0%)	67	(22.8%)		17	(13.1%)	54	(20.8%)	

Other Fruits	Low	88	(59.5%)	161	(54.4%)		75	(58.6%)	160	(61.1%)	
	High	60	(40.5%)	135	(45.6%)		53	(41.4%)	102	(38.9%)	

Low: 1-2 times/week or less, High: 3-4 times/week or more

High HbA_1c_: the subjects with no history of diabetes mellitus with HbA_1c_ values of 5.6% or more.

DM: the subjects with history of diabetes mellitus.

Healthy 1: the healthy control group for High HbA_1c_ group, Healthy 2: the healthy control group for DM group.

p : chi-square test (#: p<0.1, *: p<0.05)

Table 4 shows the odds ratios of intake frequency of fruit and vegetables for high HbA_1c_ and DM. A significantly lower odds ratio for high HbA_1c_ was observed in the high intake group of carrot and pumpkin compared with the low intake group (0.49, 95%CI: 0.29-0.85). Significantly lower odds ratio for DM was not observed between the high intake group of vegetables and fruits and the low intake group.

**Table 4.** Odds ratios (ORs) and 95% confidence intervals (CIs) of vegetables for high HbA_1c_ and diabetes mellitus

Intake frequency of vegetables or fruits		OR for high HbA_1c_		OR for DM	
		Crude OR	Adjusted OR	Crude OR	Adjusted OR
Green leaf vegetables	Low	1.00	1.00	1.00	1.00
	High	0.92(0.50-1.72)	0.96(0.50-1.83)	1.10(0.72-1.66)	1.08(0.71-1.66)

Carrot or Pumpkin	Low	1.00	1.00	1.00	1.00
	High	0.49(0.29-0.85)*	0.52(0.29-0.95)*	1.21(0.79-1.84)	1.19(0.76-1.85)

Tomato	Low	1.00	1.00	1.00	1.00
	High	0.69(0.39-1.23)	0.71(0.39-1.28)	0.84(0.55-1.28)	0.84(0.55-1.29)

Cabbage or Lettuce	Low	1.00	1.00	1.00	1.00
	High	1.19(0.58-2.45)	1.14(0.53-2.44)	1.02(0.67-1.55)	1.03(0.61-1.59)

Chinese cabbage	Low	1.00	1.00	1.00	1.00
	High	0.92(0.55-1.55)	0.94(0.55-1.60)	0.74(0.44-1.23)	0.71(0.44-1.21)

Seaweeds	Low	1.00	1.00	1.00	1.00
	High	1.02(0.55-1.90)	1.07(0.56-2.05)	0.91(0.59-1.38)	0.83(0.53-1.28)

Oranges	Low	1.00	1.00	1.00	1.00
	High	0.75(0.44-1.27)	0.87(0.50-1.52)	0.57(0.32-1.03)	0.59(0.32-1.10)

Other Fruits	Low	1.00	1.00	1.00	1.00
	High	0.87(0.53-1.43)	1.08(0.61-1.91)	1.11(0.72-1.71)	1.23(0.79-1.94)

Low: 1-2 times/week or less, High: 3-4 times/week or more

95% confidence intervals in parentheses.

Adjusted OR : Odds ratio adjusted for smoking habit and alcohol consumption.

*: p<0.05

Table 5 shows the levels of serum carotenoids, *α*-tocopherol and retinol among the study group. Serum levels of carotenoids, excluding canthaxanthin, were significantly lower in the High HbA_1c_ group than in the Healthy 1 group. Serum *β*-cryptoxanthin levels were significantly lower in the DM group than in the Healthy 2 group, although serum levels of other carotenoids, *α*-tocopherol and retinol displayed no significant differences between the DM and Healthy 2 groups. Serum levels of carotenoids, excluding canthaxanthin and *β*-cryptoxanthin, were significantly lower in the High HbA_1c_ group than in the DM group.

**Table 5.** Comparison of serum levels of carotenoids, *α*-tocopherol and retinol among the study groups

Items (*µ*mol/l)	High HbA_1c_	Healthy 1	p	DM	Healthy 2	p
*β*-carotene	0.87	1.24	***	1.14	1.21	
	(0.28-2.39)	(0.45-3.29)		(0.41-3.46)	(0.47-3.09)	

*α*-carotene	0.10	0.13	***	0.12	0.12	
	(0.04-0.22)	(0.05-0.31)		(0.05-0.32)	(0.05-0.28)	

Lycopene	0.38	0.46	*	0.50	0.45	
	(0.14-1.02)	(0.17-1.23)		(0.17-1.25)	(0.16-1.23)	

*β*-cryptoxanthin	0.20	0.26	***	0.21	0.25	*
	(0.08-0.42)	(0.10-0.63)		(0.08-0.48)	(0.10-0.59)	

Zeaxanthin & Lutein	0.97	1.17	***	1.10	1.11	
	(0.56-1.63)	(0.62-1.58)		(0.57-2.01)	(0.57-1.87)	

Canthaxanthin	0.02	0.02		0.02	0.02	
	(0.010-0.037)	(0.010-0.044)		(0.010-0.040)	(0.010-0.043)	

*α*-tocopherol	29.22	27.60		28.92	26.92	
	(19.83-46.08)	(20.24-39.21)		(20.31-42.32)	(19.28-38.14)	

Retinol	3.13	2.98		3.02	2.91	
	(2.18-4.46)	(2.13-4.05)		(2.08-4.10)	(2.00-4.05)	

High HbA1c: the subjects with no history of diabetes mellitus with HbA_1c_ values of 5.6% or more.

DM: the subjects with history of diabetes mellitus.

Healthy 1: the healthy control group for High HbA_1c_ group, Healthy 2: the healthy control group for DM group.

Data represented as geometric mean and range of 10-90% in parentheses.

p: t test (log-transformed )

*: p<0.05, ***: p<0.001

The results from the analyses conducted to compare the intake frequencies of fruit and vegetables and the serum carotenoids levels between DM and Healthy 1 group were also similar to those using Healthy 2 group as controls.

Table 6 shows the odds ratios of serum carotenoids, *α*-tocopherol and retinol for high HbA_1c_. Significantly lower odds ratios for high HbA_1c_ were observed in the high group for serum *α*- and *β*-carotene, lycopene, *β*-cryptoxanthin, and zeaxanthin & lutein than in the low group. However, the odds ratios of serum retinol and *α*-tocopherol were not significantly different between the high and low groups. Even after adjusting for confounding factors, these associations did not change.

**Table 6.** Odds ratios (ORs) and 95% confidence intervals (CIs) of serum levels of carotenoids, *α*-tocopherol and retinol for high HbA_1c_

Items		Range(*µ*mol/l)	No. of High HbA_1c_ / Healthy1	Crude odds ratio	Adjusted odds ratio^a^
*β*-carotene					
	Low	0.08-0.96	81 / 102	1.00	1.00
	Middle	0.97-1.88	42 / 100	0.53(0.33-0.84) *	0.52(0.30-0.90) *
	High	1.89-8.66	28 / 100	0.35(0.21-0.59) **	0.36(0.22-0.80) **

*α*-carotene					
	Low	0.01-0.08	75 / 111	1.00	1.00
	Middle	0.09-0.19	52 / 97	0.79(0.51-1.24)	0.94(0.56-1.57)
	High	0.20-2.63	24 / 94	0.38(0.22-0.65) *	0.51(0.27-0.96) *

Lycopene					
	Low	0.01-0.33	66 / 102	1.00	1.00
	Middle	0.34-0.69	48 / 100	0.74(0.47-1.18)	0.76(0.45-1.29)
	High	0.70-3.19	37 / 100	0.57(0.35-0.93) *	0.58(0.35-0.99) *

*β*-cryptoxanthin					
	Low	0.03-0.18	72 / 101	1.00	1.00
	Middle	0.19-0.36	54 / 100	0.76(0.48-1.19)	0.73(0.43-1.24)
	High	0.37-1.91	25 / 101	0.35(0.20-0.59) **	0.41(0.22-0.79) **

Zeaxanthin & Lutein					
	Low	0.27-0.95	71 / 101	1.00	1.00
	Middle	0.96-1.40	53 / 103	0.73(0.47-1.15)	0.66(0.39-1.09)
	High	1.41-6.67	27 / 98	0.39(0.23-0.66) *	0.37(0.20-0.60) **

Canthaxanthin					
	Low	0.010-0.019	52 / 84	1.00	1.00
	Middle	0.020-0.030	72 / 134	0.87(0.55-1.36)	1.05(0.63-1.74)
	High	0.031-0.090	27 / 84	0.52(0.30-0.90)	0.71(0.38-1.33)

*α*-tocopherol					
	Low	10.84-24.43	44 / 101	1.00	1.00
	Middle	24.44-30.61	45 / 102	1.01(0.62-1.67)	0.67(0.37-1.20)
	High	30.62-65.43	62 / 99	1.44(0.89-2.31)	0.83(0.43-1.59)

Retinol					
	Low	1.19-2.73	50 / 102	1.00	1.00
	Middle	2.74-3.35	35 / 103	0.69(0.42-1.16)	0.62(0.36-1.01)
	High	3.36-7.01	66 / 97	1.39(0.88-2.20)	0.75(0.43-1.31)

95% confidence intervals in parenthesis.

^a^: Odds ratio adjusted for smoking habit, alcohol consumption, systolic blood pressure, BMI and serum levels of total cholesterol, triglyceride and *γ*-GTP activity.

*: p<0.05, **p<0.01

A significantly lower odds ratio for DM was observed in the high group of *β*-cryptoxanthin than in the low group. No significant differences in serum other carotenoids, *α*-tocopherol or retinol were observed between the high and low groups (Table 7).

**Table 7.** Odds ratios (ORs) and 95% confidence intervals (CIs) of serum levels of carotenoids, *α*-tocopherol and retinol for diabetes mellitus

Items		Range(*µ*mol/l)	No. of DM / Healthy2	Crude odds ratio	Adjuste odds ratio^a^
*β*-carotene					
	Low	0.08-0.88	50 / 89	1.00	1.00
	Middle	0.89-1.74	45 / 91	0.87(0.53-1.43)	0.95(0.53-1.69)
	High	1.78-9.18	38 / 86	0.78(0.46-1.32)	1.16(0.60-2.25)

*α*-carotene					
	Low	0.01-0.09	45 / 87	1.00	1.00
	Middle	0.10-0.17	42 / 93	0.87(0.52-1.46)	1.08(0.61-1.93)
	High	0.18-1.20	46 / 86	1.03(0.62-1.72)	1.28(0.70-2.34)

Lycopene					
	Low	0.01-0.36	43 / 92	1.00	1.00
	Middle	0.37-0.70	43 / 87	1.01(0.61-1.69)	1.15(0.65-2.03)
	High	0.71-2.61	47 / 84	1.20(0.72-1.98)	1.30(0.74-2.31)

*β*-cryptoxanthin					
	Low	0.02-0.19	51 / 89	1.00	1.00
	Middle	0.20-0.34	52 / 85	1.09(0.61-1.69)	1.07(0.61-1.90)
	High	0.35-1.91	30 / 92	0.57(0.34-0.98)*	0.68(0.36-1.30)

Zeaxanthin & Lutein					
	Low	0.27-0.86	49 / 85	1.00	1.00
	Middle	0.87-1.26	36 / 89	0.69(0.41-1.16)	0.77(0.44-1.37)
	High	1.27-6.67	48 / 92	0.88(0.54-1.46)	0.97(0.55-1.70)

Canthaxanthin					
	Low	0.010-0.019	46 / 74	1.00	1.00
	Middle	0.020-0.029	42 / 98	0.66(0.39-1.11)	0.65(0.37-1.16)
	High	0.030-0.105	45 / 94	0.74(0.44-1.24)	0.76(0.42-1.35)

*α*-tocopherol					
	Low	10.84-24.82	33 / 93	1.00	1.00
	Middle	24.83-31.33	49 / 90	1.49(0.90-2.48)	1.70(0.92-2.99)
	High	31.34-94.11	51 / 81	1.75(1.04-2.95)	1.59(0.79-2.87)

Retinol					
	Low	0.87-2.58	41 / 89	1.00	1.00
	Middle	2.59-3.32	38 / 90	0.91(0.54-1.53)	0.68(0.38-1.23)
	High	3.33-6.53	54 / 87	1.37(0.82-2.29)	0.95(0.52-1.72)

95% confidence intervals in parenthesis.

^a^ : Odds ratio adjusted for smoking habit, alcohol consumption, systolic blood pressure, BMI and serum levels of total cholesterol, triglyceride and *γ*-GTP activity.

*: p<0.05

In addition, the results obtained using the logistic regression between DM and Healthy1 group were also similar to those using Healthy 2 group as controls.

## DISCUSSION

Few studies have reported the relationships between serum carotenoids and DM or chronic hyperglycemia. The NHANES III study revealed that serum *β*-carotene levels in subjects with impaired glucose tolerance and newly diagnosed with diabetes were lower compared to individuals with normal glucose tolerance.^13^ In addition, serum lycopene levels demonstrated an inverse relationship to glucose intolerance.^13^ In a nested case-control study in Finland,^22^ high levels of *β*-carotene and *α*-tocopherol were associated with decreased risk of non-insulin dependent DM.

In this study, the High HbA_1c_ group demonstrated significantly lower intakes of carrot and pumpkin and serum levels of serum carotenoids, such as *α*- and *β*-carotene, lycopene, *β*-cryptoxanthin, and zeaxanthin & lutein compared to the Healthy 1 group. Furthermore, significantly low odds ratios for high HbA_1c_ were observed in the high groups of *α*- and *β*-carotene, lycopene, *β*-cryptoxanthin, and zeaxanthin & lutein.

Chronic hyperglycemia leads to auto-oxidation of glucose and causes nonenzymatic glycation of proteins through Maillard’s reaction.^1^^,^^5^ In these processes, reactive oxygen species are produced.^1^ The reactive oxygen species are well known as important risk factors for cardiovascular disease^9^^,^^23^ and cancer.^9^^,^^24^ The reactive oxygen species cause pancreatic *β*-cell damage and lead to insulin pathocrinia.^5^ In an animal experimental study,^25^ hyperglycemia caused oxidative damage to the pancreatic *β*-cells of GK rats, a model of Type 2 diabetes.

Antioxidant enzymes such as superoxide dismutase and glutathione peroxidase play protective roles against the reactive oxygen species.^1^ Chronic hyperglycemia reportedly causes glycation of those enzymes and reduces these functions,^26^^,^^27^ a factor for increased oxidative stress *in vivo*.

Carotenoids such as *β*-carotene are known to protect cells from oxidative stress by quenching free radicals.^9^^,^^10^ In addition, there was significantly negative correlation between serum *β*-carotene and HbA_1c_ levels in this study subjects (r=0.119, p<0.05). Carotenoids are particularly abundant in green-yellow vegetables and fruits.^28^ Our previous report^29^ revealed that serum levels of carotenoids such as *α*- and *β*-carotene were correlated with intake frequencies of green-yellow vegetables and fruits. Low serum levels of carotenoids such as *α*- and *β*-carotene may reflect to low intake of fruit and vegetables in carotenoids. Spearman’s rank correlation coefficient between intake frequency of carrot or pumpkin, however, and serum *β*-carotene was 0.20-0.25 and those between intake frequency of other vegetables or fruits and serum other carotenoids levels were approximately 0.20.^29^ There was insufficient validity of questionnaire of dietary food intake in this study, therefore, and clearly results might not be obtained.

We suggested that individuals with high HbA1c values display lower serum carotenoids such as *α*- and *β*-carotene, due to both low intake frequencies of fruit and vegetables and increased production of the reactive oxygen species by chronic hyperglycemia.^1^

Another report showed that serum levels of vitamin C and E, which play as antioxidants, were lower in people with DM and impaired glucose tolerance.^27^ Furthermore, oral administration of large doses of *α*-tocopherol to subjects with diabetes and of ascorbic acid to non-diabetic subjects decreased glycosylated hemoglobin concentrations.^7^^,^^30^

Our results that the difference of serum *β*-carotene levels between in High HbA_1c_ group and in Healthy 1 group were approximately 30%. In the NHANES III study,^13^ the difference of serum *β*-carotene levels in subjects with impaired glucose tolerance or in subjects newly diagnosed with diabetes were 13% and 20% lower, respectively, compared to subjects with normal glucose tolerance. This difference might be affected by the difference of case and control subjects. Our case subjects were high HbA_1c_ group that was HbA_1c_ values of 5.6% or more, but subjects in the NHANES III study were classified by an oral glucose tolerance test based on the World Health Organization criteria. Our control group was healthy subjects with no history of illness, but that of NHANES III study was subjects with normal glucose tolerance including those with illness, except DM. Moreover, serum *β*-carotene levels in our study subjects were higher than in the NHANES III study subjects (geometric mean of the subjects with normal glucose tolerance: 0.425 *µ*mol/l).

In the DM group, intake frequency of oranges rich in *β*-cryptoxanthin and serum levels of *β*-cryptoxanthin were lower compared with the Healthy 2 group. Serum levels of other carotenoids such as *β*-carotene were not significantly different between DM and Healthy 2 groups.

No differences in serum levels of total cholesterol and TBARS were observed between DM and Healthy 2 groups. The percentage of ex-smokers was higher in the DM group than the Healthy 2 group. It is possible that these results reflect the effect of medical treatment, including health education, in medical institutions. Serum levels of lipid peroxidation in DM patients with good control are not elevated.^26^ We suggest that the DM group in this study included many patients with good control.

There were two limitations in this study subjects. First, the subjects in the High HbA_1c_ group were individuals with newly untreated DM or abnormal carbohydrate tolerance. It is impossible to exclude newly untreated DM from High HbA_1c_ group, because we cannot diagnose diabetes mellitus from only results of health examination. According the Japan Diabetes Society criteria,^31^ the diagnosis of diabetes can be made by fasting blood glucose levels of 126mg/dl or higher and HbA_1c_ values of 6.5% or higher. There were 12 subjects (7.9%) who fulfilled those criteria (newly DM group) in High HbA_1c_ group of this study. The same results were obtained when those subjects were excluded in analysis. Newly DM group had significantly lower levels of serum *β*-carotene than Healthy 1 group. Serum other carotenoids levels in newly DM group were lower than Healthy 1 group, but not significant (data not shown).

Second, many subjects had attended these health examinations two or more times in the present study. We therefore suggest that many subjects paid close attention to their health. Subjects in the DM group complied with health guidance and many good control patients were included.

Results from this study suggest that intake of fruit and vegetables rich in carotenoids might be a protective factor against hyperglycemia.
